# Personalized Epigenome Remodeling Under Biochemical and Psychological Changes During Long-Term Isolation Environment

**DOI:** 10.3389/fphys.2019.00932

**Published:** 2019-07-31

**Authors:** Fengji Liang, Ke Lv, Yue Wang, Yanhong Yuan, Liang Lu, Qiang Feng, Xiaolu Jing, Honghui Wang, Changning Liu, Simon Rayner, Shukuan Ling, Hailong Chen, Yumin Wan, Wanlong Zhou, Li He, Bin Wu, Lina Qu, Shanguang Chen, Jianghui Xiong, Yinghui Li

**Affiliations:** ^1^State Key Laboratory of Space Medicine Fundamentals and Application, China Astronaut Research and Training Center, Beijing, China; ^2^Lab of Epigenetics and Health Prediction, SPACEnter Space Science and Technology Institute, Shenzhen, China; ^3^Department of Medical Genetics, Oslo University Hospital, University of Oslo, Oslo, Norway

**Keywords:** Mars-500, isolation and confinement, DNA methylation, Personalized Epigenetic-Phenotype Synchronization Analysis (PeSa), long term isolation, glucose, mood state

## Abstract

It has been reported that several aspects of human health could be disturbed during a long-term isolated environment (for instance, the Mars-500 mission), including psychiatric disorders, circadian disruption, temporal dynamics of gut microbiota, immune responses, and physical-activity-related neuromuscular performance. Nevertheless, the mechanisms underlying these disturbances and the interactions among different aspects of human adaptation to extreme environments remain to be elucidated. Epigenetic features, like DNA methylation, might be a linking mechanism that explains the involvement of environmental factors between the human genome and the outcome of health. We conducted an exploration of personalized longitudinal DNA methylation patterns of the peripheral whole blood cells, profiling six subjects across six sampling points in the Mars-500 mission. Specifically, we developed a Personalized Epigenetic-Phenotype Synchronization Analysis (PeSa) algorithm to explore glucose- and mood-state-synchronized DNA methylation sites, focusing on finding the dynamic associations between epigenetic patterns and phenotypes in each individual, and exploring the underling epigenetic connections between glucose and mood-state disturbance. Results showed that DMPs (differentially methylated-probes) were significantly enriched in pathways related to glucose metabolism (Type II diabetes mellitus pathway), mood state (Long-term depression) and circadian rhythm (Circadian entrainment pathway) during the mission. Furthermore, our data revealed individualized glucose-synchronized and mood-state-synchronized DNA methylation sites, and PTPRN2 was found to be associated with both glucose and mood state disturbances across all six subjects. Our findings suggest that personalized phenotype-synchronized epigenetic features could reflect the effects on the human body, including the disturbances of glucose and mood-states. The association analysis of DNA methylation and phenotypes, like the PeSa analysis, could provide new possibilities in understanding the intrinsic relationship between phenotypic changes of the human body adapting to long-term isolation environmental factors.

## Introduction

Adaptation to an extreme environment such as long-term isolation during a human spaceflight, represents a formidable medical challenge, but it provides a unique platform for investigating human adaptation to extreme environmental changes. Mars-500, a precursor to a planned mission to Mars and the longest space flight-related isolation experiment by far, was conducted partially to collect psychological and physiological data for the effects of a long-term deep space mission (see Materials and Methods). Previously, analyses from the collected data have reported that several aspects of human health could be disturbed during a long-term isolation environment, including psychiatric disorders (Wang et al., 2014), circadian disruptions (Basner et al., 2013), temporal dynamics of gut microbiota (Turroni et al., 2017), immune responses (Yi B. et al., 2014), and physical-activity-related neuromuscular performance (Belavy et al., 2013), suggesting that human health was disturbed at multiple phenotypic aspects during the mission. However, the mechanisms underlying these disturbances were poorly explored, and the association between different aspects of the health outcome underlying adaptation to prolonged isolation and a confined environment remains to be determined.

The ability of environmental factors to shape human health involves epigenetic mechanisms that mediate gene-environment interactions. DNA methylation, comprising the heritable changes in gene structure and function that occur without a change in the nucleotide sequence, is one of the most intensively studied epigenetic modifications in mammals (Bird, 2007). Since the association between genome-wide DNA methylation and phenotype has been reported in population cohorts (Rakyan et al., 2011; Petersen et al., 2014; Doostparast Torshizi and Fazel Zarandi, 2015), twins studies (Martino et al., 2013; Jaskowiak et al., 2014; Yi H. et al., 2014) and cell studies (Lorthongpanich et al., 2013; Ziller et al., 2013; Wang and Pan, 2014)_ENREF_10, DNA methylation might be a linking mechanism which explains the involvement of environmental factors between the human genome and health outcomes. Moreover, “Longitudinal” or “temporal” factors have been reported as important factors which could drift methylation variations (Flanagan et al., 2015; Richmond et al., 2015; Simpkin et al., 2015; Grant et al., 2017; Naumova et al., 2017; Marzi et al., 2018). It is reasonable to hypothesize that epigenetic profiles (like DNA methylation) could drift during longitudinal confinement during the Mars 500 mission, and the DNA methylation change could be associated with different aspects of phenotype variations (such as physiological and psychological regulation).

In this study, as part of the Mars-500 project, we conducted a personalized dynamic interrogation of genome-wide DNA methylation profiling of six subjects at six sampling points during the simulation. Simultaneously, we measured mood state and plasma metabolic traits to investigate Psycho-Epigenome-Metabolism changes during adaptation to long term isolation. Intriguingly, we developed a PeSa algorithm to explore glucose and mood-state-synchronized DNA methylation sites, focusing on finding the dynamic associations between epigenetic patterns and phenotypes in each individual, and exploring the underling epigenetic connections between glucose and mood-state disturbances. Our data revealed individualized glucose-synchronized and mood-state-synchronized DNA methylation sites, and PTPRN2 was found to be associated with both glucose and mood state disturbances across all six subjects. It suggests that personalized phenotype-synchronized epigenetic features could reflect the effects on the human body, including the disturbances of glucose and mood states.

## Materials and Methods

### Mission Background

The Mars-500 project was conducted by the Institute of Biomedical Problems (IBMP) of the Russian Academy of Sciences (RAS) in Moscow and was aimed at simulating an interplanetary manned flight, with a particular focus on the health and working capacity of the crew members. The data collected in this study focuses on the changes observed during the three stages of simulated flight (flight to, landing at, and return from Mars). The six-member crew consisted of three Russians, one Italian, one French and one Chinese individual. All the scientific experiments, including this study protocol, were reviewed and approved by the IBMP committee on Bioethics. Before participation, all six crew members underwent a thorough clinical examination and signed an informed consent form for the long-term isolation and confinement experiment. At the beginning of the simulation experiment, the age of the crew was 32.7 ± 4.7 (range 27∼38 years) and body weight was 83.4 ± 9.2 kg (range 73.5∼99.5).

The isolation facility for the simulation experiment was located at IBMP in Moscow, Russia. The isolation facility, as well as the operation rooms, technical facilities and offices, so that the simulated space flight mission could be contained in a single building at the institute. The periods of the virtual flight to Mars throughout the 520-days of isolation were as follows.

*Period 1:* 1–11 days – flight along a spiral path in the gravitational field of Earth.
*Period 2:* 51–204 days – flight on a heliocentric trajectory to Mars.
*Period 3:* 205–243 days – flight along a spiral path in the gravitational field of Mars (“twist”).
*Period 4:* 244–272 days – flight in Mars’ orbit with descent of the 111 takeoff and landing module on the planet surface and returning to the Martian expeditionary facility. In this period a simulation of three trips with two subjects to the surface of Mars was carried out. At this time three crew members rested in the model of the Martian orbital facility observing and communicating with the crew of the takeoff and landing module.
*Period 5:* 273–309 days – flight along a spiral path in the gravitational field of Mars (“spin up”).
*Period 6:* 310–467 days – flight on a heliocentric trajectory up to the vicinity of Earth.
*Period 7:* 468–520 days – flight along a spiral path in the Earth’s gravitational field.

Food was primarily provided by companies or agencies from Russia, Europe, Korea, and China. Nutrients in the food met the recommended criteria of the World Health Organization and the Russian and American standards for crew members in the International Space Station. Food during the first half of the mission (+1 day∼+250 days) included frozen foods (56 out of 111 types of food) while the second half of the mission (+250 days∼+520d) consisted mainly of dry foods, and there were also small differences in the food providers, e.g., the food during the first half of the mission contained a certain proportion of European food, while the food during the second half of the mission included some Korean and Chinese food. During the 30 days of the simulated landing period, some crew members, including one Russian, one Italian and one Chinese member shared the diet for the landing mission, which was different than that of the other three crew members. Considering the necessary nutritional intake, all Mars500 crewmembers had three meals each day according to the arranged daily menu, which was recycled every 7 days for the first half of the mission and every 5 days for the second half of the mission.

### Biochemical and Epigenetic Assay

Peripheral whole blood cells were extracted from all six crew members before breakfast on −7, +60(+61), +120(+121), +168(+169), +249(+250), +300(+301), +360(+361), +418(+419), +510(+511), and +527 days. For each blood extraction procedure, all six crew members were equally and randomly divided into two groups on two consecutive days, respectively. The day given in brackets represents the day for the second group for each sampling event. After extraction, whole blood cells were immediately treated with EDTA anticoagulation, transferred outside of the module and centrifuged (at 3,000 rpm for 15 min at 4°C) to isolate the plasma from blood cells. Plasma parameters concentrations or counts were assayed with enzyme-linked immunosorbent assay (ELISA) kits (R&B, United States), or radio-immunological assay (RIA) kits (R&B, United States) according to the manufacturer’s instructions. DNA Methylation profiling for the blood cells were also determined using the Illumina 450k platform, and characterization of the dynamic variations in the blood cell methylome were clustered and depicted.

DNA was extracted from frozen blood samples by standard proteinase K/RNase treatment and phenol/chloroform extraction. Bisulfite modification of DNA (≥500 ng for each sample) was conducted using an EZ DNA Methylation Kit (Zymo Research) according to the manufacturer’s procedure. The Infinium Methylation 450K assay was performed according to Illumina’s standard protocol.

### HM450K Data Preprocessing

Raw intensity data (IDAT) files were imported into the R environment (version 3.4.1) and processed using the ChAMP and minfi package. All analyses were performed in R using packages available from the Bioconductor project. Probes were then removed in data preprocessing: (Wang et al., 2014) probes with a bead-count <3 in at least 5% of samples, (Basner et al., 2013) non-cg probes, (Turroni et al., 2017) probes with SNPs, (Yi B. et al., 2014) probes that align to multiple locations. Probes on the X and Y chromosomes were kept since all the subjects are male. Following this, a final data set of 412,413 probes and 37 samples (six sampling points for six subjects, and one replicate sample) remained for downstream analysis.

### Analysis of Biochemical Indicators

Repeated Measures Analysis was used to find significant changes in all indicators in this study, which is considered to be typical longitudinal data from factorial experiments. We applied a parametric method (i.e., ANOVA) to determine significance. Additionally, considering the small sample size in this study and the restrictive distributional assumptions for the use of parametric and semiparametric procedures, we also employed non-parametric rank-based methods (i.e., nparLD test, which stands for “Non-parametric Analysis of Longitudinal Data”), which is robust to outliers and exhibits competitive performance (Noguchi et al., 2012). For a specific indicator, the output of the ANOVA or nparLD test was a p-value, and an indicator was defined as significantly changed across all the sampling points during the mission if the *p* -value < 0.05 (ANOVA or nparLD test). The ANOVA test was performed by R package “stats” (version 3.1.1). The nparLD test was performed by R package “nparLD” (version 2.1), and the ANOVA-type *p* -value was taken.

### Algorithm of PeSa Analysis

Considering the longitudinal time-course of the data in the 520-day simulated flight mission, for PeSa analysis between indicators, we not only considered the traditional Pearson correlation across all sampling points, but also the similarity of the time-course curve shape by a slope-correlation, which applies the Pearson correlation to the time-course gradient of indicators. The slope correlation analysis highlights the time-course synchronization or variation amongst indicators. For a correlation between time-course indicator ***A*** = (a_1_, a_2_, …, a_i_) and ***B*** = (b_1_, b_2_, …, b_i_), where *i* is the number of sampling points, the slope correlation is performed as follows:

i. *P* -value_Corr_(***A, B***) was generated by Pearson correlation(***A, B***)
ii. A^*^ = *(a_2_,a_3_, …, a_i_)- (a_1_, a_2_, …, a_i–1_)*, and B^*^ = *(b_2_, b_3_, …, b_i_) - (b_1_, b_2_, …, b_i–1_)*
iii. *P* -value_Slope–corr_(***A, B***) was generated by Pearson correlation(A^*^, B^*^)
iv. indicator ***A*** and ***B*** was defined as significantly synchronization if *P* -value_Corr_(***A, B***) < 0.05 and *P* -value_Slope–corr_(***A, B***).

### Analysis of Mood States

The Profile of Mood States is a specific psychological questionnaire (Lindgren et al., 1999; Xu et al., 2003; Wang et al., 2014) and has been widely used to assess transient and distinct mood states in a range of different research studies, such as evaluation of individuals’ psychological conditions for occupational health and safety or under extreme environments (e.g., the expedition in Antarctic exploration; Lindgren et al., 1999; Xu et al., 2003; Wang et al., 2014). It consists of 65 adjectives that are rated by subjects on a 5-point scale (1 = lowest level, 5 = highest level). Six factors have been derived from this test, including TA, DD, FI, AH, CB, and VA.

This test was manipulated on a 14-in Lenovo laptop (1280^*^800 pixels) during confinement. Each crewmember had a unique ID and password to run this program. At the beginning of the test, the instructions were provided in three languages (English, Russian, and Chinese), the crewmembers could then begin the test. When the crewmembers completed all 65 ratings, the data were automatically exported into a zip file with the crewmember’s ID and the testing date (e.g., 5001_20100605.zip)

The standard Total Mood Disturbance (TMD) score was defined as follows.

(1) TMD=T⁢A+D⁢D+A⁢H+F⁢I+C⁢B-V⁢A

VA: Vigor-Activity; TA: Tension-Anxiety; DD: Depression-Dejection; AH: Anger-Hostility; FI: Fatigue-Inertia; CB: Confusion-Bewilderment.

## Results

### Data Design

**FIGURE 1:**
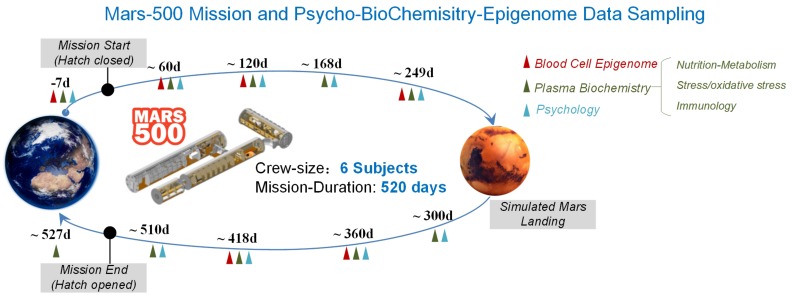
Testing schedule of simulation experiment and biochemical changes in the MARS500 mission. Schematic diagram of the Mars-500 simulation space flight mission. Vertical arrowheads represent the time points of data sampling points, which are distributed over the whole flight mission. Colored triangles showed the testing time points for psychology, plasma biochemistry and blood cell DNA methylation profiling, respectively. The timepoints occupy the whole simulation process from pre-isolation, during isolation (traveling to Mars, landing on Mars and returning back to earth) and post-isolation phases.

Over the course of this 520 days mission, we conducted personalized longitudinal DNA methylation patterns of peripheral whole blood cell profiling of six subjects across six sampling points. Multiple sampling points correspondingly synchronized with DNA methylation profiling were assigned to characterize three categories of parameters related to nutrient metabolism, stress and immunology (10 sampling points, covering six sampling points of DNA methylation profiling). Additionally, POMS scores (14 sampling points, covering six sampling points of DNA methylation profiling) were detected to investigate mood state variation during the mission (Figure 1).

### DNA Methylation Data Pre-processing

We interrogated the genome-wide DNA methylation profile using the Illumina HumanMethylation450 BeadChip (450k), which covers more than 480,000 methylation sites in the genome. The signal was represented as a beta value normalized by the Funnorm method implemented in the R package *ChAMP* and *minfi*, and batch effect was corrected by ComBat method in R package *sva* (Leek et al., 2012; Aryee et al., 2014; Morris et al., 2014) _ENREF_15. Sample quality control was performed by R package *MethylAid*. Stringent quality control steps to assess probe performance (see Materials and Methods). Since many EWAS aim to identify associations between methylation and diseases or environmental factors that have relatively small effects on the methylome (<10%), unwanted variation can be a significant problem for such studies, particularly when the number of samples is not large. To minimize the technical error and to validate its influence on biological variation, we performed replicate DNA methylation for one sample. Finally, 37 samples with data from 412,413 probes were remained for downstream analysis.

We performed replication hybridizations for one pair of the replication samples to assess sensitivity to detect biological variability across all sampling points versus technical variation. Biological variation was represented by sd_personal_(β) (standard-deviation of β-value across sampling points for each subjects) and range_personal_(β) (range of β-value across sampling points for each subjects). Technical variation was represented by an error(β) between one pair of replicates. For all pre-filtered probes, >75% probes have an error(β) less than 0.008, compared with that, >50% of probes have an sd_personal_(β) more than 0.012 and >75% of probes have a range_personal_(β) more than 0.016 (Supplementary Figure S1). Results showed that, though the effect on the epigenome is relatively small, the majority of the pre-filtered probes have biological variation beyond technical variation.

### DMPs Identification Across the Mission

**FIGURE 2:**
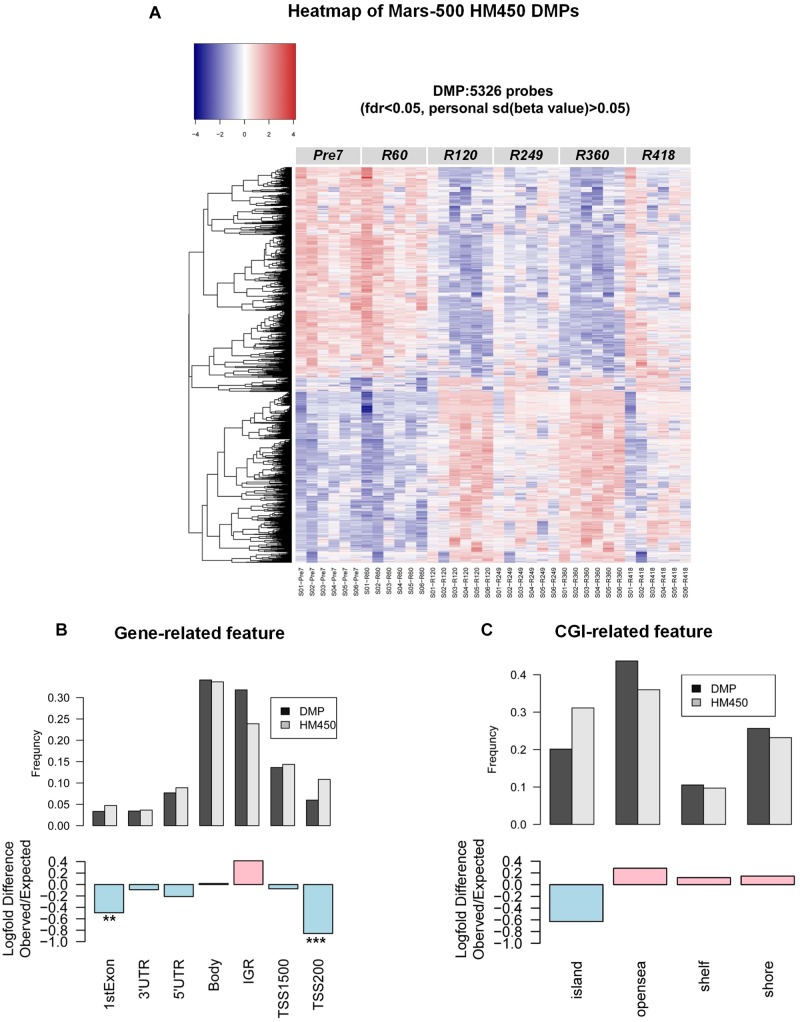
DNA methylation changes in the MARS500 mission. **(A)** Heatmap of 5326 DMPs in this study. **(B)** Enrichment of DMPs (differentially methylated-probes during the mission) by genomic location. Log-fold difference of enrichment (observed/expected frequency) in the 5326 DMPs [ANOVA fdr < 0.05, nparLD test fdr < 0.05, and personal sd(β) > 0.02] for specific genomic locations, grouped by association with genes. Pink bar indicates enrichment and light blue bar indicate depletion in the DMPs dataset. *P* -values. ^∗∗^*P* < 1 × 10–20, ^∗∗∗^*P* < 1 × 10–50. **(C)** as in **(B)**, 5326 DMPs for specific genomic locations, grouped by association with CGI. Pink bar indicates enrichment and light blue bar indicate depletion in the DMPs dataset.

To identify “truly” differential DNA methylation sites across the experiment, we added a further stringent cutoff of a sd_personal_(β) > 0.02 for all subjects in filtered probes with methylation drift far exceeding the mean error in this study. We then applied a parametric repeated measure analysis method (i.e., ANOVA) to find DMPs (differentially methylated-probes). Additionally, considering the small sample sizes in this study and the restrictive distributional assumptions for use of parametric and semiparametric procedures, we also employed non-parametric rank-based methods (i.e., nparLD test), which is robust to outliers and exhibits competitive performance. Exact 5326 DMPs were significantly changed (ANOVA fdr < 0.05, and nparLD test fdr < 0.05) (Figure 2A). Functional annotation of the DMP-associated genes was analyzed by DAVID (v6.8) (Huang da et al., 2009a,b). Results show that DMP-associated genes over-represented in platelet activation, circadian entrainment and the glucose metabolism-related pathway (Table 1 and Supplementary Tables S1–S6). In order to characterize whether DMPs were more likely to occur at specific regions in the genome, we calculated the observed/expected frequency (enrichment) of gene-related and CGI-related locations annotated in the HM450 manifest and assigned *P* -values with hypergeometric tests. The 1stExon, 3′UTR, 5′UTR, TSS1500, TSS200 and CpG-Island regions showed significantly depletion in the DMPs dataset during the mission (Figures 2B,C).

**TABLE 1:** KEGG pathway enrichment analysis for DMPs in the MARS500 mission.

**KEGG pathway**	**FDR < 0.05**			**FDR < 0.05**		
	**sd_personal_(β) > 0.02**			**sd_personal_(β) > 0.05**		
	**Top-1k genes**	**Top-2k genes**	**Top-3k genes**	**Top-1k genes**	**Top-2k genes**	**Top-3k Genes**
hsa04611:Platelet activation	3.5e-2 (0.567)	–	–	3.7e-2 (0.47)	–	–
hsa04930:Type II diabetes mellitus	2.1e-2 (0.547)	7e-3 (0.13)	1e-2 (0.107)	–	2.5e-2 (0.28)	1e-2 (0.149)
hsa04911:Insulin secretion	–	2e-3 (0.077)	4e-4 (0.014)	–	3.91e-3 (0.098)	1.2e-2 (0.268)
hsa04931:Insulin resistance	–	–	–	1.2e-2 (0.268)	3.3e-2 (0.267)	–
hsa04713:Circadian entrainment	–	3e-3 (0.07)	7e-4 (0.023)	–	–	–
hsa04730:Long-term depression	–	1.2e-2 (0.176)	–	–	–	–

*Significance of pathway enrichment was shown as “*p* -value (Benjamini)” in this table. For full analysis, see Supplementary Tables S1–S6.*

### Phenotypes Change Across the Mission

**FIGURE 3:**
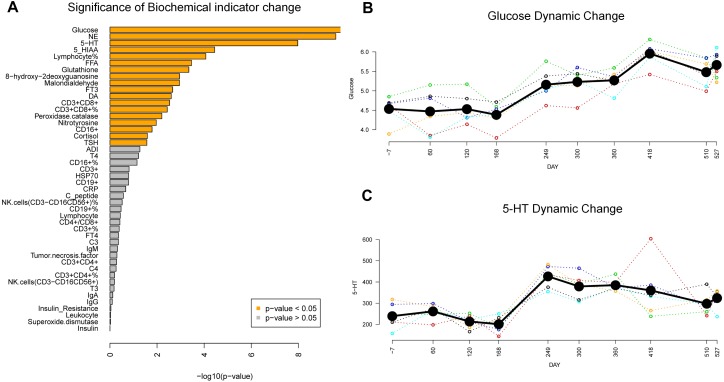
Biochemical changes in the MARS500 mission. **(A)** Significance of Biochemical indicator change during the mission. 18 indicators (orange bar) significantly changed (*P* < 0.05, ANOVA). **(B)** Glucose dynamic change pattern during the mission. Bold black line: mean value of subjects at each sampling points. Colored dash line, glucose trend for each subject. **(C)** 5-HT dynamic change pattern during the mission. Bold black line: mean value of subjects at each sampling points. Colored dash line, 5-HT trend for each subject.

The 46 blood biochemical indicators were assayed across the mission, which could be broadly grouped into three categories, related to nutrient metabolism, stress and immunology. In the DMPs identification, we applied both parametric and non-parametric repeated measures analysis, and found 18 indicators significantly changed across the mission (ANOVA *P* < 0.05, and nparLD test *P* < 0.05) (Figure 3A). Fasting blood glucose, the most significantly changed one, showed a 3-stage stepwise raising trend (Figure 3B). Following the initial average levels of 4.5 ± 0.34 mmol/L recorded before the missions start (Day-7), blood glucose levels remained stable until Day-168. After this, elevated glucose levels emerged with average levels reaching 5.2 ± 0.37 mmol/L, and within the final stage the average glucose levels reached 6.0 ± 0.37 mmol/L at Day-418. In particular, one subject exceeded 6.1 mmol/L, which is the limit for the clinical diagnostic standard for IFG, more commonly known as pre-diabetes. Glucose-related biochemical parameters such as 5-HT (Figure 3C), NE, and cortisol, though significantly changed across the mission, showed no evidence for similar glucose step-wised profiles (Supplementary Figures S2, S3). Nevertheless, other glucose-related biochemical parameters such as insulin, C-peptide, ADI, were not significantly changed. Additionally, considering that mood state and BMI (Body Mass Index) are also associated with glucose metabolism, we tracked the POMS (14 sampling points) and BMI scores (20 sampling points). Results showed that no significant changes were found in POMS-TMD and BMI score (Supplementary Figures S4, S5).

### Phenotype-Synchronized Methylation Sites

**FIGURE 4:**
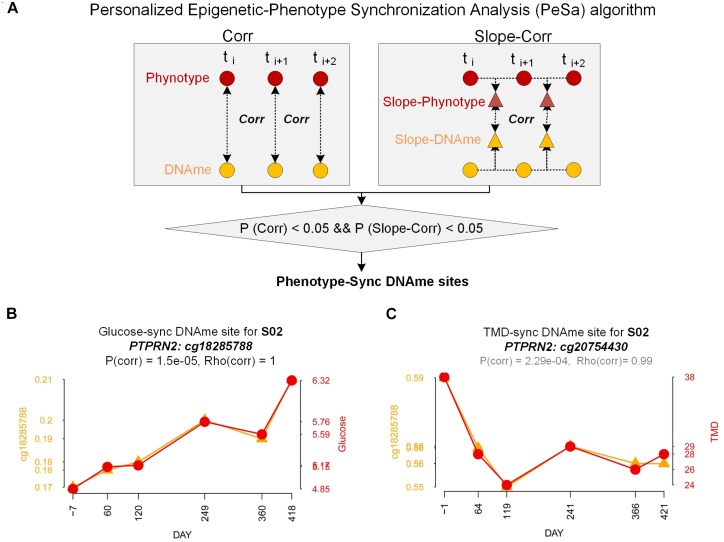
Personalized Epigenetic-phenotype Synchronization Analysis (PeSa). **(A)** Pipelines of Personalized Epigenetic-phenotype Synchronization Analysis. **(B)** Demonstration of glucose-sync DNAme site (cg18285788 at PTPRN2) for subject S02. Orange line: DNA methylation trend (beta-value). Red line: glucose trend. **(C)** Demonstration of TMD-sync DNAme site (cg20754430 at PTPRN2) for subject S02. Orange line: DNA methylation trend (beta-value). Red line: TMD trend.

From the above DNA methylation clustering results, we found that individual difference is an important factor and cannot be ignored when finding phenotype-associated epigenetic features in this study. We developed a PeSa algorithm to explore phenotype-synchronized changed DNA methylation sites (Figure 4A, Materials and Methods). By applying this method, we could find the dynamic associations between epigenetic and phenotypes in individual perspectives. In PeSa analysis, we considered not only the traditional Pearson correlation between phenotypes and epigenetics across all sampling points, but also the similarity of the time-course curve shape by a slope-correlation, which applies the Pearson correlation to the time-course gradient of indicators. In this criterium, we found “strictly” synchronized DNA methylation sites for specific phenotypes. To extend the scanning range in the epigenome, for a specific individual, we filtered all DNA methylation probes with biological variation exceeded technical variation by sd_personal_(β) > 0.01, resulting in 239566 – 274864 candidate DNA methylation sites for the six subjects. By applying PeSa analysis, we found personalized synchronized DNA methylation sites for glucose, which showed the most significantly changed biochemical indicator during the mission (Supplementary Table S7). About 829 – 1795 probes (0.35–0.74% of candidate probes) changes were found to have synchronized with glucose levels for the six individuals. For instance, probe cg18285788 (annotated in *PTPRN2)*, significantly synchronized with the glucose variation for subject S02 (Figure 4B). Furthermore, glucose-synchronized probes were annotated in gene-related features and the probes on IGR were removed, then gene covered glucose-synchronized probes were sorted according to the most consistently found numbers for the subjects. Results showed that *PTPRN2*, *PRDM16*, *KCNQ1* and *COL11A2* are glucose-synchronized genes consistently found in all six subjects (Table 2 and Supplementary Table S8). Then we performed functional annotation for the most glucose-synchronized methylation sites found in the six subjects (sorted by rho-value of traditional Pearson correlation). Results showed that, *type II diabetes mellitus* pathway *was* significantly enriched (*p* = 5.12e-4 for top-3000 genes, and *p* = 3.11e-3 for top-2000 genes) (Table 3 and Supplementary Tables S9, S10).

**TABLE 2:** Glucose-synchronized and POMS-TMD-synchronized methylation sites found in PTPRN2 gene for six subjects.

**Phenotype-sync probes (in PTPRN2)**	**Subjects**					
	**S01**	**S02**	**S03**	**S04**	**S05**	**S06**
Glucose-sync	cg19803194	cg02306654	cg06018853	cg00720339	cg00369194	cg07624226
	cg05874166	cg11315900	cg16249010	cg02660277	cg05241143	cg27629384
	cg22960901	cg12211161			cg06000610	
		cg13909612			cg07015608	
		cg18285788			cg10257673	
		cg19744528			cg24254317	
POMS-TMD-sync	cg16995768	cg05971373	cg01496696	cg18411660	cg16068431	cg03014997
	cg21280014	cg06018853	cg05064223	cg24998459	cg03071124	cg08401938
	cg24221919	cg07959864	cg07015608		cg04064735	cg09193477
		cg11900120	cg07979652		cg04498153	cg12271047
		cg20673767	cg08102838		cg04622731	cg13525062
		cg20754430	cg09042009		cg04864538	cg23655400
			cg10471944		cg07267845	cg25566285
			cg21521989		cg09845489	cg01536803
			cg21608691		cg10288307	cg05171921
					cg11251200	cg11517132
					cg11900120	cg18411660
					cg12144100	cg24093300
					cg14178347	
					cg14527439	
					cg14706245	
					cg20983647	
					cg22213057	
					cg22598247	
					cg24688143	
					cg25264630	
					cg25481630	

*For full analysis, see Supplementary Table S8.*

**TABLE 3:** KEGG pathway enrichment analysis for glucose-sync and POMS-TMD-sync genes.

**KEGG pathway**	**Glucose-sync genes**		**POMS-TMD-sync genes**	
	**Top-3k genes**	**Top-2k genes**	**Top-3k genes**	**Top-2k Genes**
hsa04930:Type II diabetes mellitus	5.12e-4 (0.01)	3.11e-3 (0.059)	0.029 (0.15)	2.11e-3 (0.062)
hsa04730:Long-term depression	–	–	1.92e-3 (0.025)	0.031 (0.237)
hsa04022:cGMP-PKG signaling pathway	1.2e-3 (0.018)	3.82e-3 (0.068)	4.27e-4 (9e-3)	4.02e-3 (0.081)
hsa04713:Circadian entrainment	2.8e-3 (0.024)	–	9.61e-4 (0.014)	–
hsa04611:Platelet activation	0.03 (0.16)	–	9.38e-4 (0.015)	1.55e-3 (0.068)
hsa05032:Morphine addiction	7.8e-3 (0.054)	–	6.88e-5 (2.41e-3)	1.74e-3 (0.058)
hsa04020:Calcium signaling pathway	1.47e-4 (0.005)	2.911e-4 (0.013)	0.018 (0.117)	–

*Significance of pathway enrichment was shown as “*p* -value (Benjamini)” in this table. For full analysis, see Supplementary Tables S11, S12.*

Considering that the crew exhibited a fluctuation of psychological adaptation similar to the third-quarter phenomenon during 520 days of confinement as previously reported (Wang et al., 2014), we also applied PeSa analysis for a mood state-related indicator POMS-TMD score. About 1018–11026 probes (0.41–4.52% of candidate probes) were found to be synchronized with and changed by POMS-TMD for the six individuals (Supplementary Table S11). For instance, probe cg20754430 (annotated in *PTPRN2)*, significantly synchronized with the POMS-TMD variation for subject S02 (Figure 4C). By way of analyzing glucose-synchronized probes, we found *PTPRN2, MAD1L1, COL11A2, NFATC1*,*RPTOR*, and *TNXB* were POMS-TMD-synchronized genes which repeated in all six subjects (Table 2 and Supplementary Table S12). Notably, *PTPRN2* covered both glucose- and POMS-TMD-synchronized probes in our finding. For most POMS-TMD-synchronized methylation sites found in the six subjects (sorted by rho-value of traditional Pearson correlation), functional annotation showed that the *long-term depression* pathway was significantly enriched (*p* = 1.92e-3 for top-3000 genes, and *p* = 0.031 for top-2000 genes). Additionally, we found that the *Circadian entrainment* pathway was also significantly enriched (*p* = 9.61e-4 for top-3000 genes) (Table 3). Evidence has shown that circadian disruption was found to be involved in psychiatric disorders (Jones and Benca, 2015), suggesting that mood-state fluctuation had an inseparable connection at least in part due to circadian disturbance in this study. Additionally, evidence based on a cell culture and depression-modeled animal study offers clues that several significantly enriched pathways have a certain relationship with long term depression, such as the cGMP-PKG signaling pathway (*p* = 4.27e-4 for top-3000 genes) (Kawaguchi and Hirano, 2013; Zhou et al., 2017), Platelet activation (*p* = 9.38e-4 for top-3000 genes) (Hufner et al., 2015), Morphine addiction (*p* = 6.88e-5 for top-3000 genes) (Naziroglu and Demirdas, 2015) and the Calcium signaling pathway (*p* = 0.02 for top-3000 genes) (Berridge, 2017; Can et al., 2017) (Supplementary Tables S13, S14).

## Discussion

Our study offers a unique perspective on human adaptation to long-term isolation, compared to traditional case/control studies. We investigated the adaptation at phenotypic, biochemical and psychological levels in both a population and individual manner. While previous analyses of data in the Mars500 experiment have reported insights of psychiatric disorders (Wang et al., 2014), circadian disruptions (Basner et al., 2013), temporal dynamics of gut microbiota (Turroni et al., 2017), immune responses (Yi B. et al., 2014), and physical-activity-related neuromuscular performance (Belavy et al., 2013), this study was focused on individualized high frequency sampling for epigenetic profiling across the mission, and exploring the dynamic linking between DNA methylation changes and different aspect of phenotypes (POMS-TMD for mood-state, and glucose for metabolism).

First, we demonstrated a novel strategy to study the relationship between epigenetic signatures and phenotypes based on long-term epigenome-phenome tracking data sets and developed the PeSa analysis method, different than traditional population case/control studies. Interindividual variance, an unignored factor in both epigenomic and phenotypic research, was also deeply explored in popular studies, especially for the small size of the cohort. For instance, POMS-TMD score fluctuations showed significant distinction among subjects (Supplementary Figure S5). Additionally, a personalized analysis has received a lot of attention in previously reported studies of the Mars-500 experiment, such as circadian and gut microbiota (Basner et al., 2013; Turroni et al., 2017). Intriguingly, in this study we developed a PeSa algorithm and traced temporal synchronous DNA methylation features, which refer to phenotypes from different aspects, including glucose for metabolism, and POMS-TMD for mood state. By applying the PeSa analysis, we could comprehensively discover the environmental-factor-induced inter-individual responses of the human body and find potential biomarkers. These are worth researching further, e.g., health risks, like metabolism or/and mood state disturbance, caused by long term isolation confinement.

Second, we found that global DNA methylation remodeling during long term isolation were evidence- supported. DMPs-associated genes were significantly enriched in *the Circadian entrainment pathway* during the mission, coinciding with previous studies which claimed that the majority of crew members experienced disturbances of sleep quality, vigilance deficits and altered sleep–wake periodicity and timing (Basner et al., 2013). Several glucose metabolism-related pathways were also significantly enriched, including *Type 2 diabetes mellitus* and *insulin signaling pathway.* Accordingly, we also found that the glucose level changed significantly during the mission. This finding is consistent with earlier studies, which show global changes in DNA methylation in association with glycemic metabolisms (Pearce et al., 2012) and insulin resistance (Zhao et al., 2012). It has also been reported that platelet activation is associated with DM and hyperglycemia (Sudic et al., 2006; Tang et al., 2011). In this study, we found that DMPs-associated genes were significantly enriched in the *platelet activation pathway*, while plasma serotonin (5-HT), a platelet-stored vasoconstrictor (Brenner et al., 2007), was also detected to be significantly changed. The association between platelet activation and major depressive disorder (MDD) was also reported in MDD patients vs. mentally healthy controls (Hufner et al., 2015). Notably, though the long-term isolation in this study did not exert the subjects into disease-states like T2D or MDD patients, similar associations among glucose-metabolism, depression and platelet activation were found. It is interesting that we found these associations from an epigenetic perspective, suggesting that these associations not only exist in clinical studies, but might possibly also occur in the process of environmental-factor-induced glucose-metabolism and mood state disorder over millions of years of adaptation in human beings to the terrestrial environment.

Third, the specific gene, *PTPRN2*, was consistently found to cover DNA methylation sites synchronously changing with glucose and POMS-TMD across all six subjects in this study. *PTPRN2*, also known as IAR, has been identified as an autoantigen in insulin-dependent DM (Schmidli et al., 1998). Evidence also substantiates that *PTPRN2* was in genetic association with bipolar disorder in the GWAS study (Curtis et al., 2011; Psychiatric GWAS Consortium Bipolar Disorder Working Group, 2011). Thus, in this study it is suggested that applying the PeSa analysis in a long-term epigenome-phenome tracking study could facilitate the discovery of biologically meaningful epigenetic signatures, which correlate with domain-crossing phenotypes, such as metabolism and mood state. Since the current study was limited in its sample size and the data set design, the verification of glucose- and POMS-TMD- synchronized DNA methylation signatures found in this study are necessary in additional long-term confinement studies, and the validation of the PeSa method is still required by applying studies based on a distinct population size, diverse environmental factors, and different time-scales of data samplings.

## Conclusion

In this study, we conducted a personalized dynamic interrogation of the genome-wide DNA methylation profiling of six subjects at six sampling points during the Mars-520 simulated mission. We utilized mood state and plasma metabolic traits to investigate Psycho-Epigenome-Metabolism changes during adaptation to long-term isolation. Our findings suggest that epigenetic features could reflect the effects of long-term isolation on the human body, such as glucose and mood state disturbances. Genes with personalized phenotype-synchronized epigenetic features could reflect the dynamic adaptation process of human adaptation to extreme environmental changes.

## Data Availability

The raw data supporting the conclusions of this manuscript will be made available by the authors, without undue reservation, to any qualified researcher.

## Ethics Statement

All the scientific experiments, including this study protocol, were reviewed and approved by the IBMP committee on Bioethics. Before the participation, all six crew members underwent a thorough clinical examination and signed an informed consent form for the long term isolation and confinement experiment.

## Author Contributions

JX and YL designed and led the data mining efforts. FL, KL, YW, HW, HC, WZ, LH, XJ, BW, and LQ performed the sample collection, mood state, biochemical, and DNA methylation measurements. FL, CL, LL, and QF conducted the data mining and bioinformatics analyses. JX, KL, YMW, YY, SC, and YL designed the experiments and interpreted the results. FL, KL, YY, SL, SR, JX, and YL wrote the manuscript.

## Conflict of Interest Statement

The authors declare that the research was conducted in the absence of any commercial or financial relationships that could be construed as a potential conflict of interest.

## Abbreviations

5-HT: 5-hydroxytryptamine
ADI: adiponectin
AH: Anger-Hostility
CB: Confusion-Bewilderment
DD: Depression-Dejection
DM: Diabetes mellitus
DMP: differentially methylated probe
FDR: false discovery rate
FI: Fatigue-Inertia
HM450: Infinium Human Methylation 450 Bead Chip array platform
IFG: impaired fasting glucose
Interval-DMR: differentially methylated region in specific Interval
NE: norepinephrine
PeSa: Personalized Epigenetic-Phenotype Synchronization Analysis
POMS: Profile of Mood States
SNP: single nucleotide polymorphism
TA: Tension-Anxiety
UTR: untranslated region
VA: Vigor-Activity.

## References

[B1] Aryee M. J. Jaffe A. E. Corrada-Bravo H. Ladd-Acosta C. Feinberg A. P. Hansen K. D. (2014). Minfi: a flexible and comprehensive bioconductor package for the analysis of infinium DNA methylation microarrays. *Bioinformatics* 30 1363–1369. 10.1093/bioinformatics/btu049 24478339 PMC4016708

[B2] Basner M. Dinges D. F. Mollicone D. Ecker A. Jones C. W. Hyder E. C. (2013). Mars 520-d mission simulation reveals protracted crew hypokinesis and alterations of sleep duration and timing. *Proc. Natl. Acad. Sci. U.S.A.* 110 2635–2640. 10.1073/pnas.1212646110 23297197 PMC3574912

[B3] Belavy D. L. Gast U. Daumer M. Fomina E. Rawer R. Schiessl H. (2013). Progressive adaptation in physical activity and neuromuscular performance during 520d confinement. *PLoS One* 8:e60090. 10.1371/journal.pone.0060090 23555896 PMC3610758

[B4] Berridge M. J. (2017). Vitamin D and depression: cellular and regulatory mechanisms. *Pharmacol. Rev.* 69 80–92. 10.1124/pr.116.013227 28202503

[B5] Bird A. (2007). Perceptions of epigenetics. *Nature* 447 396–398. 10.1038/nature05913 17522671

[B6] Brenner B. Harney J. T. Ahmed B. A. Jeffus B. C. Unal R. Mehta J. L. (2007). Plasma serotonin levels and the platelet serotonin transporter. *J. Neurochem.* 102 206–215. 10.1111/j.1471-4159.2007.04542.x 17506858 PMC3041643

[B7] Can M. S. Baykan H. Baykan O. Erensoy N. Karlidere T. (2017). Vitamin D levels and vitamin D receptor gene polymorphism in major depression. *Psychiatr. Danub.* 29 179–185. 10.24869/psyd.2017.179 28636576

[B8] Curtis D. Vine A. E. McQuillin A. Bass N. J. Pereira A. Kandaswamy R. (2011). Case-case genome-wide association analysis shows markers differentially associated with schizophrenia and bipolar disorder and implicates calcium channel genes. *Psychiatr. Genet.* 21 1–4. 10.1097/YPG.0b013e3283413382 21057379 PMC3024533

[B9] Doostparast Torshizi A. Fazel Zarandi M. H. (2015). Alpha-plane based automatic general type-2 fuzzy clustering based on simulated annealing meta-heuristic algorithm for analyzing gene expression data. *Comput. Biol. Med.* 64 347–359. 10.1016/j.compbiomed.2014.06.017 25035233

[B10] Flanagan J. M. Brook M. N. Orr N. Tomczyk K. Coulson P. Fletcher O. (2015). Temporal stability and determinants of white blood cell DNA methylation in the breakthrough generations study. *Cancer Epidemiol. Biomark. Prev.* 24 221–229. 10.1158/1055-9965.EPI-14-0767 25371448

[B11] Grant C. D. Jafari N. Hou L. Li Y. Stewart J. D. Zhang G. (2017). A longitudinal study of DNA methylation as a potential mediator of age-related diabetes risk. *Geroscience* 39 475–489. 10.1007/s11357-017-0001-z 29159506 PMC5745220

[B12] Huang da W. Sherman B. T. Lempicki R. A. (2009a). Bioinformatics enrichment tools: paths toward the comprehensive functional analysis of large gene lists. *Nucleic Acids Res.* 37 1–13. 10.1093/nar/gkn923 19033363 PMC2615629

[B13] Huang da W. Sherman B. T. Lempicki R. A. (2009b). Systematic and integrative analysis of large gene lists using DAVID bioinformatics resources. *Nat. Protoc.* 4 44–57. 10.1038/nprot.2008.211 19131956

[B14] Hufner K. Kandler C. Koudouovoh-Tripp P. Egeter J. Hochstrasser T. Stemer B. (2015). Bioprofiling of platelets in medicated patients with depression. *J. Affect. Disord.* 172 81–88. 10.1016/j.jad.2014.09.029 25451399

[B15] Jaskowiak P. A. Campello R. J. Costa I. G. (2014). On the selection of appropriate distances for gene expression data clustering. *BMC Bioinform.* 15:S2. 10.1186/1471-2105-15-S2-S2 24564555 PMC4072854

[B16] Jones S. G. Benca R. M. (2015). Circadian disruption in psychiatric disorders. *Sleep Med. Clin.* 10 481–493. 10.1016/j.jsmc.2015.07.004 26568124

[B17] Kawaguchi S. Y. Hirano T. (2013). Gating of long-term depression by Ca2+/calmodulin-dependent protein kinase II through enhanced cGMP signalling in cerebellar Purkinje cells. *J. Physiol.* 591 1707–1730. 10.1113/jphysiol.2012.245787 23297306 PMC3624847

[B18] Leek J. T. Johnson W. E. Parker H. S. Jaffe A. E. Storey J. D. (2012). The sva package for removing batch effects and other unwanted variation in high-throughput experiments. *Bioinformatics* 28 882–883. 10.1093/bioinformatics/bts034 22257669 PMC3307112

[B19] Lindgren K. N. Masten V. L. Tiburzi M. J. Ford D. P. Bleecker M. L. (1999). The factor structure of the profile of mood states (POMS) and its relationship to occupational lead exposure. *J. Occup. Environ. Med.* 41 3–10. 10.1097/00043764-199901000-00002 9924714

[B20] Lorthongpanich C. Cheow L. F. Balu S. Quake S. R. Knowles B. B. Burkholder W. F. (2013). Single-cell DNA-methylation analysis reveals epigenetic chimerism in preimplantation embryos. *Science* 341 1110–1112. 10.1126/science.1240617 24009393

[B21] Martino D. Loke Y. J. Gordon L. Ollikainen M. Cruickshank M. N. Saffery R. (2013). Longitudinal, genome-scale analysis of DNA methylation in twins from birth to 18 months of age reveals rapid epigenetic change in early life and pair-specific effects of discordance. *Genome Biol.* 14:R42. 10.1186/gb-2013-14-5-r42 23697701 PMC4054827

[B22] Marzi S. J. Sugden K. Arseneault L. Belsky D. W. Burrage J. Corcoran D. L. (2018). Analysis of DNA methylation in young people: limited evidence for an association between victimization stress and epigenetic variation in blood. *Am. J. Psychiatry* 175 517–529. 10.1176/appi.ajp.2017.17060693 29325449 PMC5988939

[B23] Morris T. J. Butcher L. M. Feber A. Teschendorff A. E. Chakravarthy A. R. Wojdacz T. K. (2014). ChAMP: 450k chip analysis methylation pipeline. *Bioinformatics* 30 428–430. 10.1093/bioinformatics/btt684 24336642 PMC3904520

[B24] Naumova O. Y. Dozier M. Dobrynin P. V. Grigorev K. Wallin A. Jeltova I. (2017). Developmental dynamics of the epigenome: a longitudinal study of three toddlers. *Neurotoxicol. Teratol.* 66 125–131. 10.1016/j.ntt.2017.12.006 29247702

[B25] Naziroglu M. Demirdas A. (2015). Psychiatric disorders and TRP channels: focus on psychotropic drugs. *Curr. Neuropharmacol.* 13 248–257. 10.2174/1570159x13666150304001606 26411768 PMC4598437

[B26] Noguchi K. Gel Y. R. Brunner E. Konietschke F. (2012). nparLD: an r software package for the nonparametric analysis of longitudinal data in factorial experiments. *J. Stat. Softw.* 50 1–23.25317082

[B27] Pearce M. S. McConnell J. C. Potter C. Barrett L. M. Parker L. Mathers J. C. (2012). Global LINE-1 DNA methylation is associated with blood glycaemic and lipid profiles. *Int. J. Epidemiol.* 41 210–217. 10.1093/ije/dys020 22422454 PMC3304536

[B28] Petersen A. K. Zeilinger S. Kastenmuller G. Romisch-Margl W. Brugger M. Peters A. (2014). Epigenetics meets metabolomics: an epigenome-wide association study with blood serum metabolic traits. *Hum. Mol. Genet.* 23 534–545. 10.1093/hmg/ddt430 24014485 PMC3869358

[B29] Psychiatric Gwas Consortium Bipolar Disorder Working Group (2011). Large-scale genome-wide association analysis of bipolar disorder identifies a new susceptibility locus near ODZ4. *Nat. Genet.* 43 977–983. 10.1038/ng.943 21926972 PMC3637176

[B30] Rakyan V. K. Down T. A. Balding D. J. Beck S. (2011). Epigenome-wide association studies for common human diseases. *Nat. Rev. Genet.* 12 529–541. 10.1038/nrg3000 21747404 PMC3508712

[B31] Richmond R. C. Simpkin A. J. Woodward G. Gaunt T. R. Lyttleton O. McArdle W. L. (2015). Prenatal exposure to maternal smoking and offspring DNA methylation across the lifecourse: findings from the avon longitudinal study of parents and children (ALSPAC). *Hum. Mol. Genet.* 24 2201–2217. 10.1093/hmg/ddu739 25552657 PMC4380069

[B32] Schmidli R. S. Colman P. G. Cui L. Yu W. P. Kewming K. Jankulovski C. (1998). Antibodies to the protein tyrosine phosphatases IAR and IA-2 are associated with progression to insulin-dependent diabetes (IDDM) in first-degree relatives at-risk for IDDM. *Autoimmunity* 28 15–23. 10.3109/08916939808993841 9754810

[B33] Simpkin A. J. Suderman M. Gaunt T. R. Lyttleton O. McArdle W. L. Ring S. M. (2015). Longitudinal analysis of DNA methylation associated with birth weight and gestational age. *Hum. Mol. Genet.* 24 3752–3763. 10.1093/hmg/ddv119 25869828 PMC4459393

[B34] Sudic D. Razmara M. Forslund M. Ji Q. Hjemdahl P. Li N. (2006). High glucose levels enhance platelet activation: involvement of multiple mechanisms. *Br. J. Haematol.* 133 315–322. 10.1111/j.1365-2141.2006.06012.x 16643434

[B35] Tang W. H. Stitham J. Gleim S. Di Febbo C. Porreca E. Fava C. (2011). Glucose and collagen regulate human platelet activity through aldose reductase induction of thromboxane. *J. Clin. Invest.* 121 4462–4476. 10.1172/JCI59291 22005299 PMC3204848

[B36] Turroni S. Rampelli S. Biagi E. Consolandi C. Severgnini M. Peano C. (2017). Temporal dynamics of the gut microbiota in people sharing a confined environment, a 520-day ground-based space simulation, MARS500. *Microbiome* 5:39. 10.1186/s40168-017-0256-8 28340597 PMC5366131

[B37] Wang Y. Jing X. Lv K. Wu B. Bai Y. Luo Y. (2014). During the long way to mars: effects of 520 days of confinement (Mars500) on the assessment of affective stimuli and stage alteration in mood and plasma hormone levels. *PLoS One* 9:e87087. 10.1371/journal.pone.0087087 24695321 PMC3973648

[B38] Wang Y. Pan Y. (2014). Semi-supervised consensus clustering for gene expression data analysis. *BioData Min.* 7:7. 10.1186/1756-0381-7-7 24920961 PMC4036113

[B39] Xu C. Zhu G. Xue Q. Zhang S. Du G. Xi Y. (2003). Effect of the Antarctic environment on hormone levels and mood of Chinese expeditioners. *Int. J. Circumpolar Health* 62 255–267. 10.3402/ijch.v62i3.17562 14594200

[B40] Yi B. Rykova M. Feuerecker M. Jager B. Ladinig C. Basner M. (2014). 520-d Isolation and confinement simulating a flight to Mars reveals heightened immune responses and alterations of leukocyte phenotype. *Brain Behav. Immun.* 40 203–210. 10.1016/j.bbi.2014.03.018 24704568

[B41] Yi H. Bo C. Song X. Yuan Y. (2014). Initial points selection for clustering gene expression data: a spatial contiguity analysis-based approach. *Biomed. Mater. Eng.* 24 3709–3717. 10.3233/BME-141199 25227086

[B42] Zhao J. Goldberg J. Bremner J. D. Vaccarino V. (2012). Global DNA methylation is associated with insulin resistance: a monozygotic twin study. *Diabetes* 61 542–546. 10.2337/db11-1048 22210312 PMC3266395

[B43] Zhou X. Y. Zhang F. Ying C. J. Chen J. Chen L. Dong J. (2017). Inhibition of iNOS alleviates cognitive deficits and depression in diabetic mice through downregulating the NO/sGC/cGMP/PKG signal pathway. *Behav. Brain Res.* 322 70–82. 10.1016/j.bbr.2016.12.046 28077315

[B44] Ziller M. J. Gu H. Muller F. Donaghey J. Tsai L. T. Kohlbacher O. (2013). Charting a dynamic DNA methylation landscape of the human genome. *Nature* 500 477–481. 10.1038/nature12433 23925113 PMC3821869

