# Supplementary material for: Personalized Epigenome Remodeling Under Biochemical and Psychological Changes During Long-Term Isolation Environment
Source: Front Physiol. 2019 Jul 31;10:932. doi: 10.3389/fphys.2019.00932 (PMC6684777; doi:10.3389/fphys.2019.00932)
Supplement: Supplementary file 1 [file Data_Sheet_1.docx]

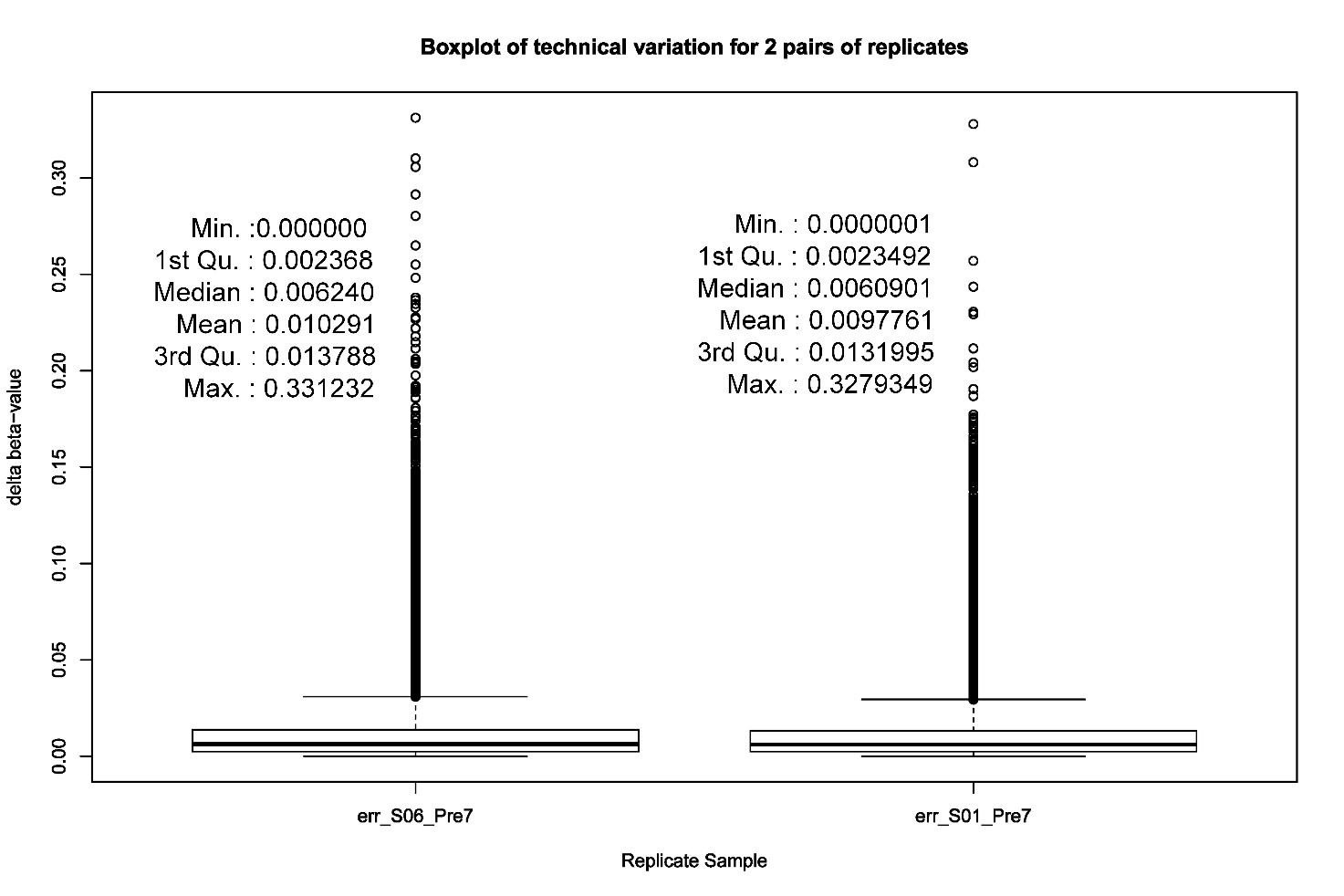


**Figure S1. Boxplot of technical variation for replicates**

For all pre-filtered probes, >75% probes have error(β) less than 0.013


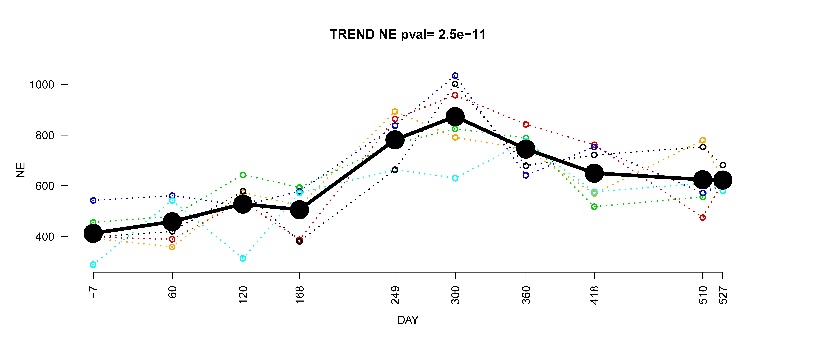


**Figure S2.** **Norepinephrine(NE) dynamic change pattern in the MARS500 mission.**

Norepinephrine significantly changed during the mission(ANOVA P=2.5e-11). Bold black line, mean value of subjects at each sampling points. Colored dash line, Norepinephrine trend for each subject.


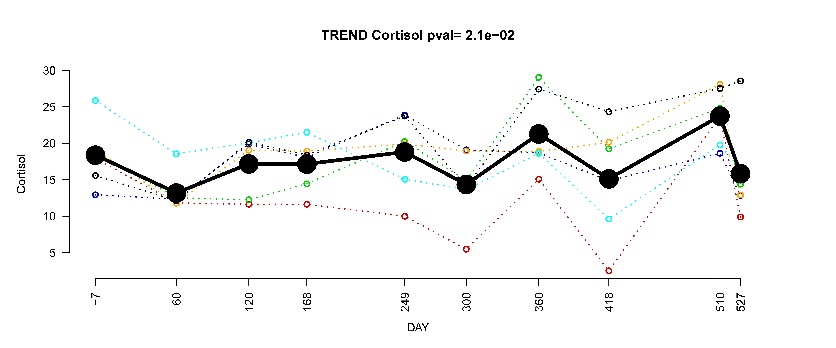


**Figure S3.** **Cortisol dynamic change pattern in the MARS500 mission.**

Cortisol significantly changed during the mission(ANOVA P=0.021). Bold black line, mean value of subjects at each sampling points. Colored dash line, Cortisol trend for each subject.


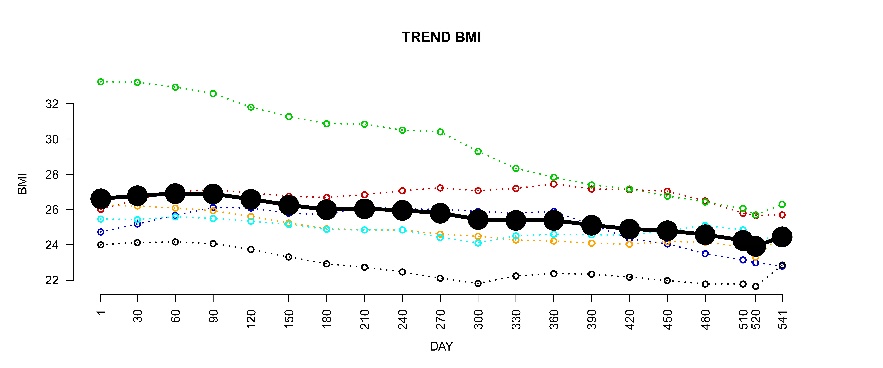


**Figure S4. BMI dynamic change pattern in the MARS500 mission.**

Cortisol did not significantly changed during the mission. Bold black line, mean value of subjects at each sampling points. Colored dash line, BMI trend for each subject.


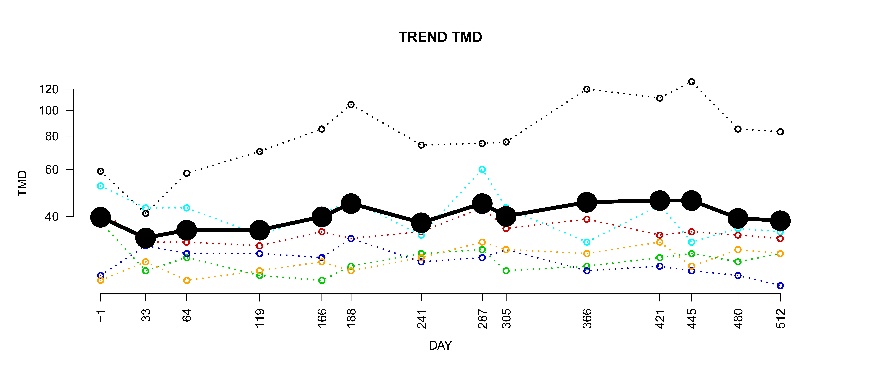


**Figure S5. POMS-TMD dynamic change pattern in the MARS500 mission.**

POMS-TMD did not significantly changed during the mission. Bold black line, mean value of subjects at each sampling points. Colored dash line, POMS-TMD trend for each subject.
